# Relief of Preintegration Inhibition and Characterization of Additional Blocks for HIV Replication in Primary Mouse T Cells

**DOI:** 10.1371/journal.pone.0002035

**Published:** 2008-04-30

**Authors:** Jing-xin Zhang, Gretchen E. Diehl, Dan R. Littman

**Affiliations:** 1 The Kimmel Center for Biology and Medicine of the Skirball Institute, New York University School of Medicine, New York, New York, United States of America; 2 Departments of Microbiology and Pathology, New York University School of Medicine, New York, New York, United States of America; 3 Howard Hughes Medical Institute, New York University School of Medicine, New York, New York, United States of America; National Cancer Institute, United States of America

## Abstract

Development of a small animal model to study HIV replication and pathogenesis has been hampered by the failure of the virus to replicate in non-primate cells. Most studies aimed at achieving replication in murine cells have been limited to fibroblast cell lines, but generating an appropriate model requires overcoming blocks to viral replication in primary T cells. We have studied HIV-1 replication in CD4^+^ T cells from human CD4/ CCR5/Cyclin T1 transgenic mice. Expression of hCD4 and hCCR5 in mouse CD4^+^ T cells enabled efficient entry of R5 strain HIV-1. In mouse T cells, HIV-1 underwent reverse transcription and nuclear import as efficiently as in human T cells. In contrast, chromosomal integration of HIV-1 proviral DNA was inefficient in activated mouse T cells. This process was greatly enhanced by providing a secondary T cell receptor (TCR) signal after HIV-1 infection, especially between 12 to 24 h post infection. This effect was specific for primary mouse T cells. The pathways involved in HIV replication appear to be PKCθ−, CARMA1-, and WASp-independent. Treatment with Cyclosporin A (CsA) further relieved the pre-integration block. However, transcription of HIV-1 RNA was still reduced in mouse CD4^+^ T cells despite expression of the hCyclin T1 transgene. Additional post-transcriptional defects were observed at the levels of Gag expression, Gag processing, Gag release and virus infectivity. Together, these post-integration defects resulted in a dramatically reduced yield of infectious virus (300–500 fold) after a single cycle of HIV-1 replication. This study implies the existence of host factors, in addition to those already identified, that are critical for HIV-1 replication in mouse cells. This study also highlights the differences between primary T cells and cell lines regarding pre-integration steps in the HIV-1 replication cycle.

## Introduction

A small animal model for HIV infection and pathogenesis would be invaluable for basic research as well as for vaccine development. Humanized mice can be used for this purpose. The xenotransplant models include severe combined immunodeficiency (SCID) mice transplanted with human fetal thymus or liver cells (SCID-Hu (Thy/Liv)), SCID mice transplanted with human peripheral blood lymphocytes (Hu-PBL-SCID) and *Rag^−/−^γ_c_^−/^* ^−^ mice reconstituted with human CD34^+^ stem cells [1], [2]. Although these mice have been useful for *in vivo* HIV-1 pathogenesis studies, as they have aspects of the human immune system, each model has its limitations. B cells fail to develop in SCID-Hu (Thy/Liv) mice, and in CD34^+^ stem cell reconstituted mice, abnormal interactions between human TCR and mouse MHC lead to defects in T cell development [1], which represents a serious problem for most of the xenotransplant models.

Various cell types in the immune system contribute to the initiation, progression and prevention of HIV-1 induced disease. Dendritic cells (DCs) and macrophages, for example, are required for the initiation of adaptive immunity, but potentially also harbor viruses and thus contribute to enhancing infection with HIV-1 [3], [4]. To understand the role of the immune system in HIV pathogenesis, it is necessary to have an intact host immune system in small animals infectable with HIV-1. In humans, combinations of CD4 and CXCR4 or CCR5 can mediate cell entry of HIV-1. Cells from rats transgenic for human CD4 and human CCR5 were susceptible to HIV-1 entry [5], [6]. In this rat model, primary macrophages and microglia supported some productive HIV-1 replication. However, rat T cells were unable to support virus spreading due to putative post-transcriptional blocks [5].

Some progress has been made in developing mouse models for HIV-1. Mice that express HIV transgenes have been generated using both the full length provirus and individual components of the HIV-1 genome including Nef, Tat, LTR and Env [7], [8], [9], [10]. Some mouse strains expressing single HIV proteins developed symptoms of AIDS such as wasting and CD4^+^ T cell depletion. However, full length HIV-1 RNAs are transcribed inefficiently in mouse cells. hCyclin T1 interacts with HIV Tat protein in a species-restricted manner to enhance RNA transcription and processing [11]. In JRCSF (R5 tropic HIV-1) and hCyclin T1 double transgenic mice, increased HIV-1 expression correlated with CD4^+^ T cell depletion [12]. In addition to hCyclin T1, hCD4 and hCCR5 or hCXCR4 are necessary for HIV-1 infection of mouse cells. Transgenic mice that express these human genes have been generated and reported [13], [14]. However, these genes alone are not sufficient to make mice susceptible to HIV-1. No virus spreading was observed *in vivo* for either hCD4/CCR5 or hCD4/CXCR4 transgenic mice [13], [14].

Studies searching for additional factors involved in species-specific restriction of HIV replication have mainly focused on cell lines such as NIH 3T3 and A9 cells [15], [16], [17]. NIH 3T3 cells expressing hCD4/CCR5/Cyclin T1 supported HIV-1 entry and integration. Virus assembly was reported to be a major post-integration block, which could be relieved in mouse-human heterokaryons [18]. A9-based somatic cell hybrid lines containing human chromosome 2 can efficiently release infectious virus [17]. The host factors required for viral release in these systems have not yet been identified. Several groups have tried to overcome the assembly/release block by altering the virus, e.g. mutation of matrix protein to enhance membrane targeting, over-expression of Gag to increase intracellular p24 level, replacing MA of HIV-1 with that of Murine Leukemia Virus (MLV), inhibition of HIV-1 RNA over-splicing, and use of the alternative RNA transport element CTE [19], [20], [21], [22], [23], [24]. We found that some lines of 3T3 cells could support efficient Gag processing and virus release, while other lines could not. Because of these discrepancies, we were concerned that 3T3 cells may not be an appropriate model for studying virus release in mouse cells. Moreover, a mouse model for HIV replication and pathogenesis will require that T lymphocytes can be infected with the virus. It is therefore critical that we have a clear understanding of how HIV replication is affected in primary mouse T cells.

HIV-1 infection in transformed mouse T cell lines was reported recently [25]. In that study, immortalized mouse T cell lines that expressed hCD4 and hCCR5 were found to support normal HIV-1 entry, but reverse transcription and nuclear import were reduced as compared to NIH 3T3 cells or human T cell lines. In the current study, we have compared the replication of HIV-1 in primary human and mouse T cells. We found that viral entry, reverse transcription, and nuclear import of HIV-1 are as efficient in primary mouse T cells as in human T cells. Although chromosomal integration of HIV-1 proviral DNA was inefficient in mouse T cells pre-activated with anti-CD3 and anti-CD28, this block was substantially relieved by providing a secondary TCR signal after HIV-1 infection. Using mutant mice, we found that this signal is independent of the pathways involving PKCθ, CARMA1 (CARD 11) and WASp. Treatment with CsA, which significantly enhances HIV-1 infection in owl monkey cells [26], [27], [28], additionally relieved the pre-integration block in mouse T cells.

Although we were able to overcome blocks in the HIV replication cycle in murine cells up to proviral integration, we observed defects in transcription, despite expression of the hCyclin T1 transgene, and after transcription at the levels of Gag expression, Gag processing and Gag release. Furthermore, infectivity of virus released from mouse T cells was lower than that of virus released from human T cells. These defects subsequent to HIV integration contributed in aggregate to a 300–500-fold reduction in the titer of virus released from mouse T cells as compared to human T cells.

## Results

### Primary T cells from hCD4/CCR5 transgenic mice support efficient virus entry

**Figure 1:**
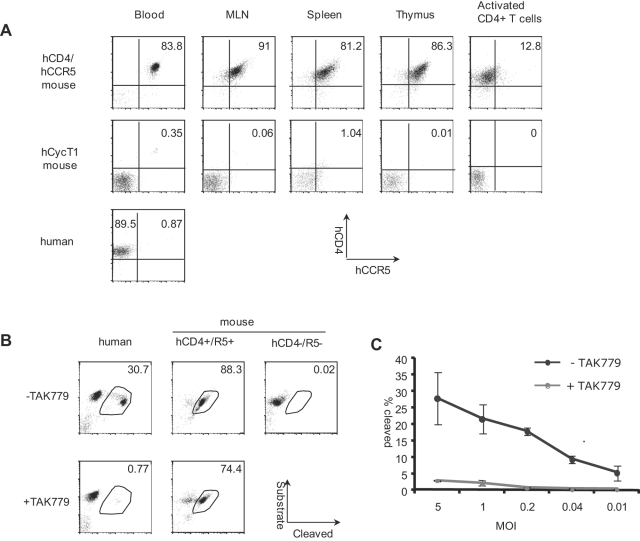
Primary CD4^+^ T cells from hCD4/hCCR5 transgenic mice support R5 HIV-1 entry. (A) Single cell suspensions prepared from mouse blood, mesenteric lymph node, spleen and thymus were analyzed by flow cytometry for the expression of human CD4 and human CCR5. Plots shown were gated on mouse CD4^+^ T cells. Mouse CD4^+^ T cells activated by plate-bound anti-CD3/28 were also stained for hCD4/hCCR5expression. hCyclin T1 transgenic mice served as negative controls. CD4^+^ T cells from human PBMCs were also analyzed for comparison. (B) Resting human and mouse CD4^+^ T cells were infected with JRCSF viruses containing Vpr-β-lactamase at MOI of 1. Cells were then loaded with CCF2-AM and incubated at 18–20°C for 2 h (mouse) or overnight (human) before fixation and flow cytometry analysis. FITC channel was used to detect the substrate and DAPI channel for cleaved product. TAK-779 (1 µM) was used to inhibit CCR5 mediated entry. Cells from non-transgenic mice were used as negative control. (C) CD4^+^ T cells from hCD4/CCR5 mice were activated with plate-bound anti-CD3/28 and analyzed as in panel B. All data represented three independent experiments.

We used transgenic mice in which expression of human CD4 and CCR5 was directed by the human *CD4* regulatory sequences (see Materials and Methods [5], [12]), in contrast to the Lck promoter used in published hCD4/CCR5 transgenic mice [13]. In humans, CD4 is expressed in T cells, macrophages and DCs. To characterize the cellular expression profiles of hCD4 and hCCR5 in these mice, we performed flow cytometry analysis by co-staining for mouse CD4, hCD4, and hCCR5. Both transgenes were highly expressed on mouse CD4^+^ T cells in all organs examined, including blood, mesenteric lymph nodes, spleen, and thymus (Figure 1A). Mouse bone marrow derived DCs also expressed hCD4/CCR5 (data not shown). hCCR5 was expressed at a much higher level on T cells from transgenic mice than on human T cells. Activation of mouse T cells using plate-coated anti-CD3 and -CD28 antibodies induced marked down-regulation of hCCR5 from the cell surface (Figure 1A and Discussion).

To measure virus entry, we utilized the Vpr-β-lactamase assay [29]. Purified mouse and human resting CD4^+^ T cells were infected at different multiplicities of infection (MOIs) with JRCSF (R5 HIV-1) containing Vpr-β-lactamase. Infected cells were then loaded with CCF2-AM. The cleavage of CCF2-AM by β-lactamase was monitored by flow cytometry using the FITC and DAPI channels, for un-cleaved substrate and cleaved products, respectively. At MOI of 1, 30% of human T cells supported virus entry, which was completely blocked by the CCR5 inhibitor TAK-779. For resting mouse CD4^+^ T cells, entry was more efficient, with about 90% of mouse cells supporting entry (Figure 1B). Treatment with TAK-779 had only a modest effect, most likely due to the extremely high level of CCR5 expression at the surface of T cells in the transgenic mice. Since activation down-regulates hCCR5 expression, we tested the ability of TAK-779 to inhibit HIV-1 entry into activated mouse CD4^+^ T cells. Under these conditions, 20% of the cells were infected, and TAK-779 effectively blocked entry (Figure 1C). There was no virus entry in control experiments with cells from hCD4/hCCR5-negative mice. Thus, R5 virus can efficiently enter mouse T cells expressing human CD4 and CCR5. Due to the different levels of hCCR5 between human and transgenic mouse T cells, we chose to use Vesicular Stomatitis Virus G protein (VSVG)-pseudotyped virus in subsequent experiments. Entry of VSVG-pseudotyped virus was similar in human and mouse activated CD4^+^ T cells as shown in Figure S1 and reported previously [25].

### Primary mouse T cells support efficient reverse transcription

We next evaluated the efficiency of reverse transcription following entry of HIV-1 into mouse T cells. To reduce DNA carry-over from plasmid used for transfection, virus-containing supernatants were treated with benzonase [30], which greatly decreased contamination of the viral prep with DNA, and had no effect on infectivity (Figure S2).

**Figure 2:**
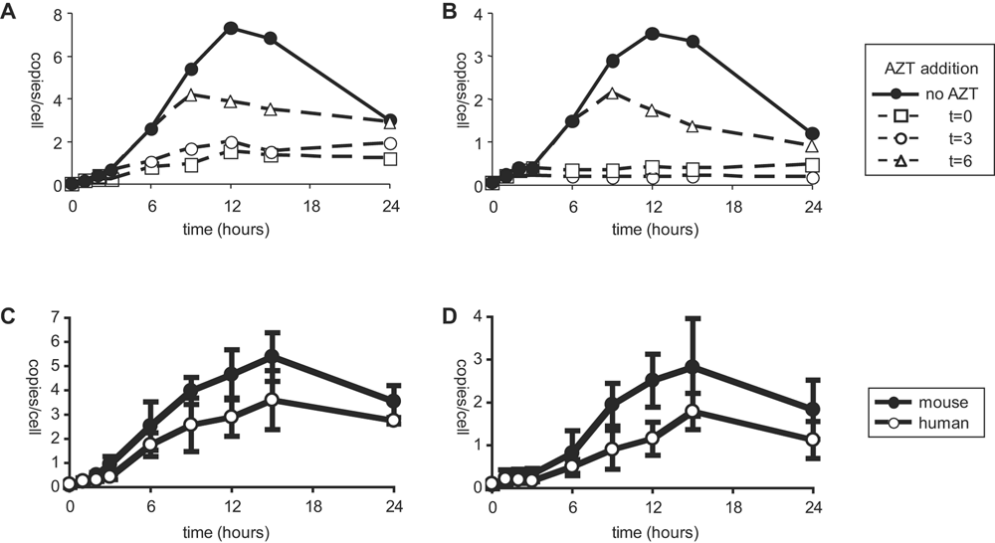
Primary CD4^+^ T cells from hCyclin T1 mice support efficient reverse transcription of HIV-1. (A) Kinetics of early reverse transcription products in mouse T cells. MACS purified CD4^+^ T cells from three individual hCyclin T1 mice were activated for 36 h by anti-CD3/28. Cells were then spin infected with benzonase-treated VSVG-pseudotyped HIV-GFP (MOI = 2). 3 h post infection, cells were washed to remove virus and replated on anti-CD3/28. At different time points, cells were collected, washed and frozen down. DNA was isolated by phenol extraction and Real Time PCR analysis was preformed. To show the de novo formation of reverse transcription products, AZT (15 µM) was added at different time points and cells were collected as above starting from the next time point (Dashed lines). Real Time-PCR was performed using primers and probes specific for 18S rRNA (for cell number) and R-U5 (for early reverse transcription product). The copy number of reverse transcription product was then normalized to cell number and displayed as copy number per cell. (B) Kinetics of late reverse transcription products. Samples from panel A were subjected to Real Time-PCR using primers and probes specific for U5-Gag. (C, D) Activated CD4^+^ T cells from three mice were infected and analyzed for early (panel C) and late reverse transcription (panel D) products as described in panel A. For comparison, PHA activated human PBMC CD4^+^ T cells from three donors were infected and analyzed in parallel. All values represent the arithmetic means ±SD. Data represent two independent experiments, each with cells from three human donors or three mice.

The kinetics of HIV-1 reverse transcription were studied in activated mouse T cells at a MOI of 2 (Figure 2A). The results were similar at higher MOI (data not shown). Early and late reverse transcription products were monitored by real-time PCR with primers specific for U5 and U5-Gag. Mouse T cells were activated for 36 h, collected and frozen down at different time points post-infection. AZT was used in control cultures to confirm that the signal was reverse transcriptase dependent. AZT was added at different time points to inhibit reverse transcription and samples were collected starting from the next time point. As shown in Figure 2A, reverse transcription started soon after virus infection and peaked 12–15 h post-infection. After that, HIV-1 DNA began to be degraded. Late reverse transcription products followed the same kinetics (Figure 2B). We and others [31], [32] found similar kinetics in human cells (Figure 2C and 2D). We next compared the copy number of reverse transcription products between human and mouse primary T cells. Cell numbers were calculated based on real-time PCR for the gene encoding 18S rRNA, using a sequence common to both human and mouse (see Materials and Methods). The copy number was then normalized to cell number and was displayed as copy number per cell. Reverse transcription products were about 50% higher in mouse than in human T cells (Figures 2C and 2D). However, this increase was not statistically significant. At the peak of reverse transcription of HIV-1 (MOI = 2), each activated mouse T cell contained approximately 5 copies of early reverse transcription product and 3 copies of late reverse transcription product. We conclude that reverse transcription proceeded as efficiently in mouse T cells as in human T cells.

### Primary T cells from hCyclin T1 transgenic mice support efficient nuclear import (formation of 2-LTR circles) but have a defect in integration

A byproduct of the process by which the HIV pre-integration complex enters the nucleus and gives rise to an integrated provirus is the formation of 2-LTR circles. 2-LTR circles are formed inside the nucleus and have been used as an indicator for HIV-1 nuclear entry [33].

**Figure 3:**
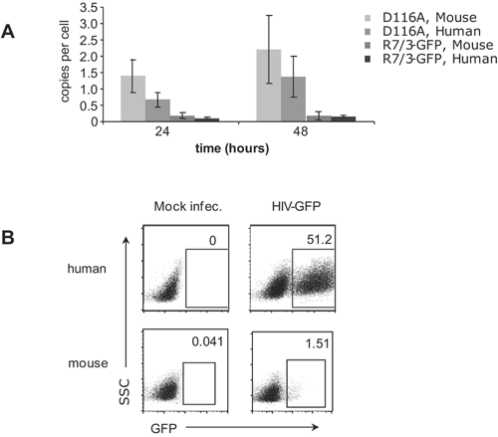
Primary CD4^+^ T cells from hCyclin T1 mice support efficient nuclear import of HIV-1 but less efficient integration. (A) CD4^+^ T cells from hCyclin T1 transgenic mice and human donors were activated and infected with VSVG-pseudotyped R7/3-GFP at MOI of 0.3 or its D116A integrase mutant virus with similar amount of RT as described in Materials and Methods. At 24 and 48 h post infection, cells were collected and subjected to real-time PCR specific for 2LTR circles. (B) CD4^+^ T cells from hCyclin T1 transgenic mice were activated and infected with VSVG-pseudotyped HIV-GFP at MOI of 2 as described in Materials and Methods. Cells were removed from anti-CD3/28 antibody at 3 h post infection. Infection was assessed by GFP expression using flow cytometry at 48 h post-infection. For comparison, activated human CD4^+^ T cells were infected and analyzed in parallel. All data represented three independent experiments.

We found no difference in the level of 2-LTR circles formed in human and mouse T cells after infection with HIV-1 (R7/3-GFP) (Figure 3A). At MOI of 0.3, there is about 0.1–0.15 copy of 2-LTR circles in each activated cell. In further support of the detection of 2-LTR circles, the signals were increased about 8–10 fold when D116A integrase mutant virus in the same R7/3 backbone was used (Figure 3A). To study virus integration more directly, we monitored the expression of GFP after infection of mouse and human T cells with HIV-GFP, a virus whose *nef* gene was replaced with *gfp*. GFP is expressed from the multiply-spliced HIV RNA. The multiply-spliced RNAs are exported from the nucleus independently of the Rev-RRE system, which is thought to be inefficient in 3T3 cells [21]. In the presence of hCyclin T1, HIV-GFP virus has been used to study blocks to HIV-1 infection in 3T3 cells [15], [16]. Expression of GFP therefore indicates successful integration, although it can be influenced by other factors such as LTR promoter activity and mRNA stability. For this reason, we have also employed a PCR-based analysis of integrated proviruses in some experiments as described below, but this was not feasible for comparing mouse and human cells.

Using HIV-GFP (VSVG) as an indicator virus, successful integration took place in 30-50% of CD4^+^ T cells in PHA-activated human PBMC. When murine T cells were stimulated with anti-CD3/28 and infected under conditions similar to those for human T cells, without secondary TCR signals, infection was very inefficient, with only about 1.5% of cells becoming infected (Figure 3B). Under these conditions, mouse T cells are therefore far less efficient than human T cells in supporting virus integration.

### Secondary TCR signals permit efficient HIV-1 infection of primary murine T cells

While optimizing conditions for maximum HIV-1 infection of primary mouse T cells, we found that pre-activation of mouse T cells was not sufficient to support HIV replication, but when secondary TCR signals were provided, much of the pre-integration block was relieved. We sought to understand the role of T cell signaling in the ability of mouse cells to support infection with HIV.

**Figure 4:**
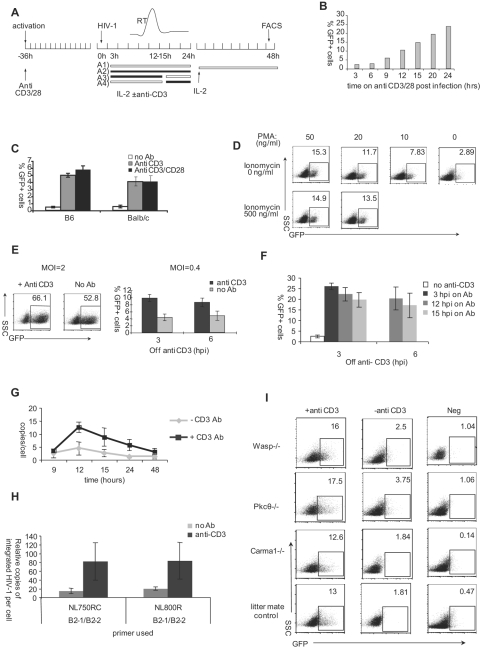
Signals from CD3 are important for HIV-1 infection in activated primary mouse T cells. The scheme for this experiment is shown in (A). MACS purified CD4*^+^* CD25*^−^* T cells were activated by anti-CD3/28 for 36 h before spin-infection with VSVG-pseudotyped HIV-GFP. Infected cells were then subjected to different culture conditions. Between 3 and 24 h post infection, cells were cultured either A1) without anti-CD3; A2) with anti-CD3; A3) with anti-CD3 for a specified period of time before removal of anti-CD3; or A4) off anti-CD3 for a specified period of time before being placed on anti-CD3. Filled bars show the presence of anti-CD3 while open bars show the absence. At 24 h post infection, all cells were cultured with mIL-2 alone. Infection was assessed by GFP expression using flow cytometry at 48 h post-infection. (B) Activated CD4^+^ T cells from hCyclin T1 transgenic mice were subjected to the analysis shown in panel A condition 3. Infected mouse T cells were incubated on anti-CD3/28 antibody first and then removed from antibody at different time point as indicated on the x-axis. (C) Activated CD4^+^ T cells from wild type C57/BL6 (B6) and Balb/c mice were infected with HIV-CMV-GFP (VSVG) at MOI of 2. After infection, cells were provided with anti-CD3/28 (black bar), anti-CD3 (grey bar) or no antibody (open bar). (D) Analysis was as in panel A condition 2, except that PMA and ionomycin were used in place of anti-CD3. (E) Analysis was done as in panel B except that MLV was used. (F) Analysis as in panel in panel A condition 4. At 3 h or 6 h post-infection, cells from hCyclin T1 mice were transferred to wells without anti-CD3. At 12 h or 15 h post-infection, these cells were transferred back to wells with anti-CD3. As controls, cells were treated under condition A1 and A2 at 3 h post infection. (G) CD4^+^ T cells from hCyclin T1 transgenic mice were infected with HIV-GFP (VSVG). Cells were taken off anti-CD3/28 at 3 h post-infection and transferred to wells without or with anti-CD3 as in panel A conditions A1 and A2. At different time points indicated on the X-axis, cells were collected and subjected to Real Time-PCR specific for U5-Gag as described in the legend to Figure 2B. (H) CD4^+^ T cells from hCyclin T1 transgenic mice were infected with HIV-GFP (VSVG). Cells were taken off anti-CD3/28 at 3 h post-infection and transferred to wells without or with anti-CD3 as in panel A conditions A1 and A2. At two days post infection, cells were collected and subjected to two–round PCRs, as described in Materials and Methods, to quantify the integrated proviruses. (I) CD4^+^ T cells from *Wasp* ^−/−^, *Pkcθ* ^−/−^ and *Carma1* ^−/−^ mice were activated with anti-CD3/28 in the presence of 100 U/ml mIL-2. After infection with HIV-CMV-GFP (VSVG), cells were provided with secondary anti-CD3 or no antibody as shown as in panel A conditions 1 and 2. All data represented three independent experiments.

Primary CD4^+^ cells from hCyclin T1 transgenic mice were purified and stimulated as described above. We then infected the activated mouse T cells with HIV-GFP (VSVG) at a MOI of 2 (Figure 4A). Three hours post-infection, the cells were washed to remove virus. Infected cells were then subjected to different culture conditions. Between 3 and 24 h post infection, cells were cultured either 1) without anti-CD3; 2) with anti-CD3; 3) with anti-CD3 for a specified period of time before removal of anti-CD3; or 4) off anti-CD3 for a specified period of time before being placed on anti-CD3. IL-2 was maintained in all culture conditions. At 24 h post-infection, the cells were transferred to a new well with mIL-2 alone. Cells were cultured for an additional 24 h before analysis by flow cytometry for infection efficiency.

HIV-1 infection efficiency in primary mouse T cells correlated well with the duration of secondary TCR stimulation after HIV infection (Figure 4B). Only 2% of cells were infected if the cells were removed from antibody at 3 h post-infection. Infection level increased linearly with the incubation time on plate-bound antibodies and reached a maximum of 25% after 24 h (Figure 4B). The infection efficiency did not increase further if cells were stimulated beyond this 24 h window (data not shown). Addition of anti-CD28 did not further increase mouse T cell infectivity (Figure 4C)

The effect of secondary stimulation was initially observed in human cyclin T1 transgenic mice. To determine if the activation signals were also necessary in primary T cells from wild type mice, we used an HIV-1 construct in which Cytomegalovirus (CMV) promoter-driven *gfp* replaced *nef*. While this virus (HIV-CMV-GFP) gave similar result to HIV-GFP virus in primary T cells from hCyclinT1 transgenic mice, it bypassed the need for hCylin T1 in mouse T cells. As shown in Figure 4C, in wild type B6 or Balb/c mice activation signals were also necessary for maximum infection, although this was lower than observed in the mixed background of the transgenic mice. Removal of anti-CD3 and CD28 after HIV-1 infection reduced the percentage of GFP*^+^* T cells 5–10-fold to 0.5–1%. Treatment with anti-CD28 or IL-2 was neither necessary nor sufficient for enhanced HIV-1 infection. We further tested if phorbol myristate acetate (PMA) and ionomycin could replace anti-CD3 in promoting HIV replication. Mouse T cells were pre-activated with anti-CD3 and anti-CD28. After HIV-1 infection, PMA and ionomycin were added instead of anti-CD3. As shown in Figure 4D, treatment with PMA alone fully recapitulated the role of anti-CD3.

To determine if the effect of TCR signaling was unique to HIV-1, we treated mouse CD4^+^ T cells in the same way during infection with MLV. At high MOI, infection efficiency was similar with or without the secondary TCR signal (Figure 4E). At low MOI, removal of the anti-CD3 antibody at 3 h post-infection resulted in a 50% reduction in infection (Figure 4E). The secondary TCR signal therefore enhances infection of murine T cells with both MLV and HIV-1, but appears to have a greater effect upon infection with HIV-1.

To further characterize the requirement for TCR signaling, we infected activated mouse T cells and re-plated them without anti-CD3 stimulation. At the peak of reverse transcription (12 to 15 h post-infection), we re-plated the cells on anti-CD3 (condition (4) in Fig. 4A). Antibody was removed at 24 h post infection and cells were assayed at 48 h post-infection. Under these conditions, 70–80% of the maximum infection efficiency was obtained, as compared to 7–10% if CD3 stimulation was not provided (Figure 4F).

To establish which stage of the HIV replication cycle was enhanced by TCR signaling, we analyzed reverse transcription under the various stimulation conditions. Late reverse transcription products were decreased about 2–3 fold in the absence of secondary stimulation (Figure 4G), but the reduction was more modest than the 10-fold effect in infection efficiency. To measure the difference in integrated provirus, a two round amplification strategy was employed, with the first round using primers specific for HIV-1 Gag and SINE B2 sequences [34], [35] and the second round using primers and probes specific for HIV-1 U5. Integrated provirus levels were reduced 5–8 fold in the absence of a secondary TCR signal (Figure 4H). As pre-activated T cells are actively engaged in cell proliferation (at least once per 24 h) and we did not find consistent down-regulation of 2-LTR circles (data not shown), we do not believe that nuclear import of HIV-1 DNA was affected in the absence of the secondary TCR signal. These results suggest that the activation signals are important for both reverse transcription and other pre-integration steps (see Discussion). It has been reported that duration of anti-CD3/28 and IL-2 signaling is important for efficient HIV-1 infection of human T cells [36]. Our results indicate that activation of primary mouse T cells prior to infection with HIV is not sufficient for viral replication, and that secondary TCR signals are required for post-entry events.

Ligation of the TCR/CD3 complex activates several distinct signaling pathways, including the NF-κB pathway that is dependent on PKC-θ and CARMA1. To determine whether this pathway has a role in promoting steps in HIV replication, we examined infection of CD4^+^ T cells from mice deficient in PKC-θ or CARMA1. Primary T cells from both strains of mutant mice supported HIV-1 infection as long as secondary TCR signals were provided (Figure 4I). Thus, signaling pathways involved in efficient integration of the HIV-1 provirus are independent of these upstream effectors of NF-κB activation. Wiskott-Aldrich Syndrome protein (WASp)-mediated cytoskeleton reorganization is another pathway downstream of the TCR and is important for immune synapse formation [37]. We obtained a similar result with T cells from WASp-deficient mice (Figure 4I) indicating that this pathway is not involved in HIV-1 replication.

### The role of secondary TCR signaling in HIV-1 infection is specific for primary mouse T cells

**Figure 5:**
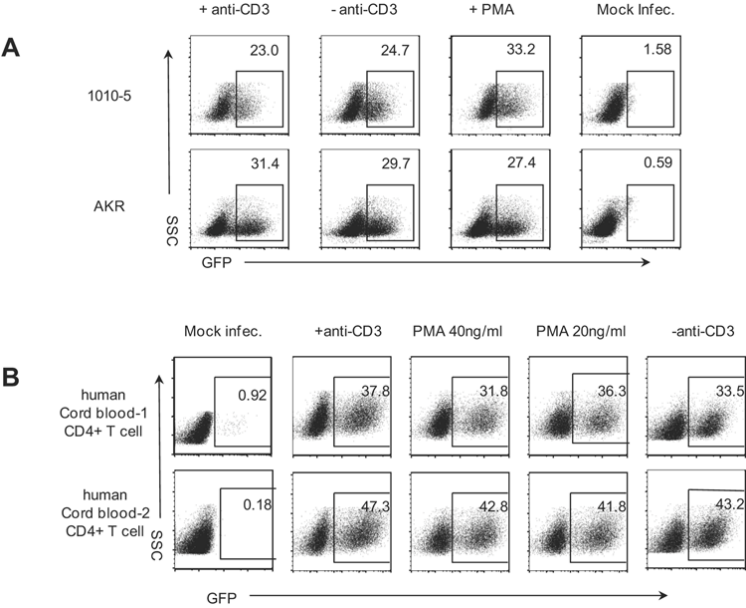
Role of secondary TCR signals is specific for mouse primary T cells. (A) mouse T cell lines 1010-5 and AKR were infected with HIV-CMV-GFP (VSVG) at MOI of 2. Three hours post infection, cells were transferred to wells with coated anti-CD3, no antibody, or PMA. Twenty-four hours post infection, cells were transferred to new wells with IL-2 alone. FACS analysis for GFP was done 48 h post infection. (B) CD4^+^ T cells from two human cord blood donors were activated with anti-CD3/28 for 36 h as described in Materials and Methods. Cells were infected and analyzed as in panel A.

We wished to determine whether a secondary TCR signal also promotes HIV-1 infection of mouse T cell lines and primary human T cells, as it does with primary mouse T cells. Infection of mouse T cell lines 1010-5 and AKR with HIV-CMV-GFP (VSVG) at MOI of 2 did not require the secondary TCR signals provided by anti-CD3 or PMA (Figure 5A). Similar results were obtained using two other mouse T cell lines (TA3 or 166, data not shown).

The majority of mouse T cells from 6–8 week old mice are naïve whereas the majority of T cells from human PBMCs are memory T cells. For a better comparison with mouse T cells, we isolated CD4^+^ T cells from human cord blood, as most of these cells are naïve. As shown in Figure 5B, secondary treatment with PMA or anti-CD3 had no effect on infection of primary human T cells. In addition, we obtained similar results when total CD4^+^ T cells from human PBMCs were used (data not shown).

### CsA further relieves the pre-integration block in primary mouse T cells

**Figure 6:**
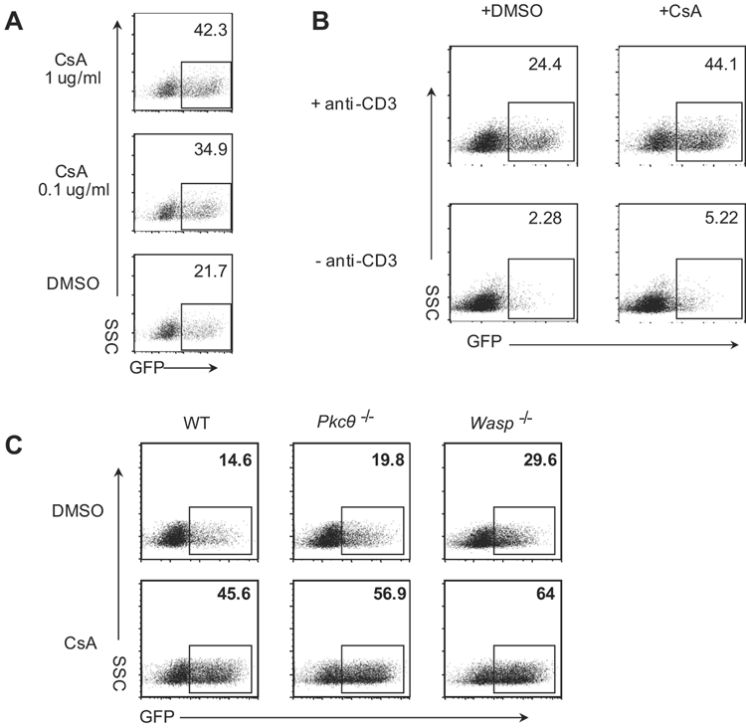
CsA further sensitizes the TCR effect on the pre-integration block in mouse T cells. (A) CD4^+^ T cells from hCyclin T1 transgenic mice were activated and infected with HIV-GFP (VSVG). Three hours post infection, cells were transferred to wells coated with anti-CD3 together with 0.1 µg/ml CsA, 1 µg/ml CsA or DMSO control. Twenty-four hours post infection, cells were washed twice with PBS and transferred to new wells with IL-2 alone. FACS analysis for GFP was done 48 h post infection. (B) Analysis was as in panel A except that at 3 h post infection cells were transferred to wells coated with or without anti-CD3, together with 0.1 µg/ml CsA. (C) CD4^+^ T cells from wild type, *Pkcθ* ^−/−^ and *Wasp* ^−/−^ mice were activated and infected with HIV-CMV-GFP (VSVG). Three hours post infection, cells were transferred to wells coated with anti-CD3, together with 0.1 µg/ml CsA, Cells were then analyzed as in panel A.

In the course of our experiments, we found that Cyclosporin A, when added 3 h post-infection, increased infection efficiency from 21% (DMSO) to 35% at 0.1 µg/ml CsA and 42% at 1 µg/ml CsA (Figure 6A). However, CsA could not substitute for TCR signals, but instead amplified the effect of TCR signals (Figure 6B). In the absence of TCR signals, CsA only modestly increased the infection efficiency from 2% to 5%. We further tested the effect of CsA on *Wasp* ^−/−^ and *Pkcθ* ^−/−^ mice. As shown in Figure 6C, the TCR signaling pathways that require these proteins are not involved in the mechanism of CsA mediated enhancement of HIV-1 infection.

### Primary T cells from hCyclin T1 transgenic mice have defects in HIV-1 transcription and translation

**Figure 7:**
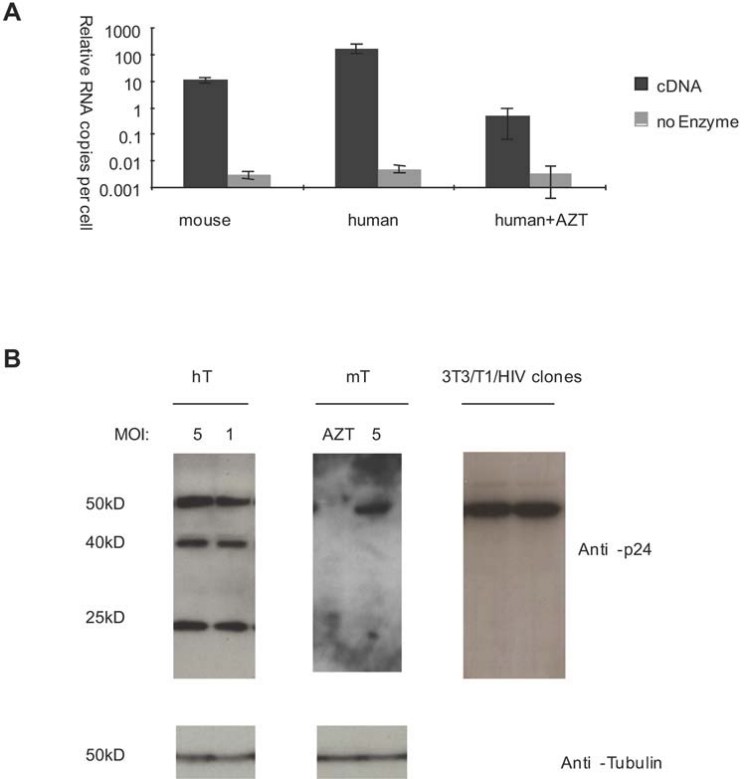
Analysis of HIV-1 RNA and proteins in infected primary mouse T cells. Primary CD4^+^ T cells from hCyclin T1 mice (mT) or human PBMCs (hT) were infected with VSVG-pseudotyped HIV-GFP at MOI of 2 in the presence or absence of AZT. Forty-eight hours later, cells were collected. (A) Total RNA was extracted from infected cells. Infection in the presence of AZT served as negative control for de novo expression of RNA. cDNA formation in the absence of reverse transcription enzyme served as negative control for contaminating DNA. Real Time-PCR was performed using primers and probes specific for 18S rRNA and R-U5 (HIV). The ratio of HIV to 18S rRNA was further normalized by infection efficiency to show the relative copy numbers of HIV RNAs per infected cell. hCyclinT1 mice gave an average of 20% infection efficiency, while human T cells gave an average of 50%. (B) Total cellular proteins were analyzed on 12% acrylamide gels and subjected to western blot analysis with anti-p24 monoclonal antibody. Endogenous α-tubulin was analyzed on the same membrane as a loading control. As a comparison, stable clones derived from 3T3/Cyclin T1 infected with HIV-GFP (VSVG) were analyzed in parallel.

The relief of the pre-integration block in primary mouse T cells enabled us to further characterize the post-integration steps in the HIV-1 replication cycle. The expression of hCyclin T1 enhances the transcription of HIV-1 RNA in primary mouse T cells [12]. When infected with HIV-GFP (VSVG), in which GFP is driven by the LTR promoter, there were very few detectable GFP positive T cells in wild type mice as compared to 15–25% in hCyclin T1 transgenic mice (data not shown). To establish whether the expression of hCyclin T1 was sufficient to relieve the transcription block in mouse T cells, we further analyzed the level of total HIV-1 RNAs in hCyclin T1 transgenic T cells as compared to human T cells by real-time PCR using primers specific for U5 sequences in the LTR (Figure 7A). Single round infection with HIV-1 (VSVG) was performed in primary mouse and human T cells. The U5 RNAs were normalized to the 18S rRNA and compared between total human and mouse cells. After normalization to infection efficiency, about 10 fold more HIV-1 RNAs were detected in infected human T cells than mouse T cells.

**Figure 8:**
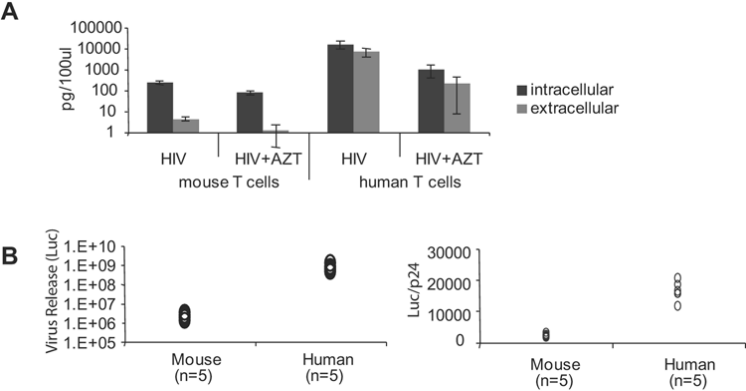
Primary CD4^+^ T cells from hCyclin T1 mice do not release virus efficiently. Activated CD4^+^ CD25^−^ T cells were infected with VSVG-pseudotyped HIV-GFP or JRCSF at MOI of 2. Three hours post-infection, cells were washed and plated on anti-CD3 with mIL-2. Twenty-four hours post-infection, cells were treated with pronase as described in Material and Methods. Infected cells were incubated at 37°C for an additional 24 h and checked by flow cytometry to assess viability. Cells and supernatants were collected after centrifugation. For comparison, activated human CD4^+^ T cells were analyzed in parallel. (A) p24 levels in cell lysates and supernatants after infection with HIV-GFP (VSVG). (B) Infectivity of virus released from cells infected with JRCSF (VSVG) was measured by adding supernatant to Tzmbl reporter cell lines. At 16 h after infection, AZT and TAK-779 were added to Tzmbl to prevent a second round of infection. Firefly luciferase assays were performed at 48 h post-infection. Luciferase signals in mock infection control were subtracted from those in infected cells. The data represented experiments using cells from five human donors or five mice. p24 levels in the supernatant (by ELISA) were used for normalization.

Subsequent steps in the HIV-1 replication cycle were further examined. Human T cells express full-length Gag proteins and efficiently process them (Figure 7B). In mouse T cells infected with an efficiency of 20–25%, expression of Gag was reduced approximately 70-fold compared to human T cells (Figure 8A) and Gag remained unprocessed (Figure 7B).

### Primary T cells from hCyclin T1 transgenic mice have defects in virus release and infectivity

To measure the efficiency of p24 release, the ratio of extracellular to intracellular p24 in HIV-infected mouse and human T cells was determined by ELISA. CD4^+^ T cells were activated for 36 h and spin infected with HIV-GFP (VSVG). Because mouse CD4^+^ T cells express and release low levels of p24, small amounts of residual input virus can lead to a false signal of viral release. To avoid this contaminating signal, the cells were treated with pronase to remove virus bound to the outside of the cell after infection. Additionally, cell death could lead to release of p24 into the medium, so cell viability was carefully monitored. We excluded any experiments in which there were fewer than 70-80% live cells at 2 d post infection. We could consistently achieve this when using highly purified CD4^+^ mouse T cells that were depleted of CD25*^+^* cells. The release of p24 was reduced in mouse T cells as compared to human cells. The ratio of extracellular to intracellular p24 averaged 0.015 for mouse T cells versus 0.4 for human T cells (Figure 8A).

To measure virus infectivity, we used a reporter cell line (Tzmbl), which is a HeLa cell line stably transfected with LTR-luciferase and hCD4/CXCR4/CCR5 [38]. Mouse and human T cells were infected with JRCSF (VSVG) and the supernatants were used to infect Tzmbl cells. Mouse T cells released 300–500-fold lower infectious virus than human T cells. After normalization to the level of p24, virus released from mouse cells had 6–10 fold lower infectivity than that from human T cells (Figure 8B).

### Primary T cells from hCyclin T1 transgenic mice have a defect in virus budding

**Figure 9:**
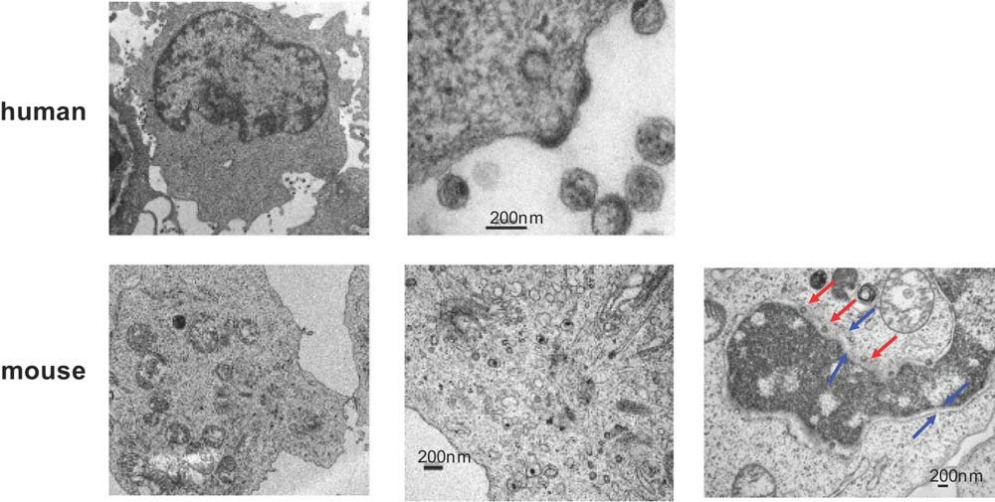
Defective virus budding in HIV infected mouse T cells. Electron microscopy of infected human (upper panel) and mouse (lower panel) T cells. On the left, low magnification of infected cells; in the middle, higher magnification of infected cells; on the right (for mouse T cells), aberrant budding of VLP into nuclear membranes is shown. Red arrows indicate assembling viral particles, while blue arrows indicate inner and outer nuclear membranes.

To view the assembly and budding of HIV, electron microscopy was performed for mouse and human T cells infected with HIV-1. The process of virus budding and release can be observed at the plasma membrane in human T cells (Figure 9, upper panels). Interestingly, viruses were not released from the entire plasma membrane. Instead, release was localized to a few discrete areas. Virus budding through the multi-vesicular body (MVB) was not detected.

No virus was detected budding from the plasma membrane in mouse T cells (Figure 9, lower panels), but some particles were observed budding into the nuclear membrane. In other cells, viruses were observed to assemble and bud randomly into or from intracellular membranes (Figure 9, lower panels).

## Discussion

We have studied HIV-1 replication in primary mouse T cells and compared the efficiency of each step in mouse cells with that in primary human T cells. Expression of hCD4, CCR5 and hCyclin T1 in mouse T cells removed the block to HIV entry and greatly enhanced viral transcription. In this study, we relieved the block prior to integration by providing TCR signals and Cyclosporin A after HIV-1 infection. However, multiple additional blocks were found to exist in primary mouse T cells. These blocks together prohibit release of high titers of infectious HIV-1 sufficient for virus spreading.

### Virus Entry

Transgenic constructs containing enhancers, promoter, and silencer of the h*CD4* gene enable high expression of hCD4 and hCCR5 on the surface of mouse CD4^+^ T cells, macrophages and dendritic cells in the transgenic mice used in this study. There are very few cells that are hCD4 or hCCR5 positive but mouse CD4 negative (Data not shown). While this is a good system to study virus entry, CCR5 is expressed at a much higher level on all mouse CD4^+^ T cells, in contrast to human T cells, in which it is expressed primarily on memory rather than naïve T cells [39]. Thus, the transgenic mouse cells were more permissive than human cells for virus entry. This transgenic model may hence lead to more severe T cell depletion if the other blocks to HIV-1 replication can be solved, and a better model to use may be hCCR5 knock-in mice.

The expression level of hCCR5 was down regulated by activation using coated anti-CD3/28 antibodies. This is consistent with previous studies showing that anti-CD28 down-regulates CCR5 in human T cells, inhibiting R5 tropic HIV-1 infection [40]. However, in mouse cells, degradation rather than synthesis of CCR5 RNA may be affected, as hCCR5 expression was not driven by the endogenous promoter. CCR5 down-regulation resulted in reduction in entry of HIV-1 into activated mouse T cells. This may not be a problem *in vivo* as HIV-1 can enter resting cells, in which reverse transcription is initiated. Subsequent steps of the HIV life cycle can then be completed upon T cell activation [41], [42]. We also note that hCCR5 staining with antibody did not correlate well with the efficiency of hCCR5 mediated virus entry. For example, a much lower percentage of human T cells can be stained with hCCR5 antibody, while they supported 30% of virus entry. This could be due to the need for few hCCR5 molecules to obtain virus entry.

### Reverse Transcription

Using HIV-1 pseudotyped with VSVG to normalize entry in mouse and human T cells, we found that HIV-1 reverse transcription had the same kinetics in the two species. Reverse transcription products peaked around 12–15 h, which was followed by down-regulation of virus DNA induced by an unknown mechanism. From these data, we are able to conclude that there is no block to reverse transcription in mouse cells. These conclusions are supported by a recent publication which also found no block to reverse transcription in primary mouse T cells [43]. However, reduction of reverse transcription products was observed in mouse T cell lines [25]. This may be because of the use of high MOI of viruses or if mouse T cell lines behave differently from primary T cells. The differences between primary mouse T cells and T cell lines may be due to genetic background or to transcriptional changes that occurred upon transformation.

### Nuclear import and integration

We found similar levels of 2-LTR circles in HIV-infected mouse and human T cells, consistent with equivalent nuclear import of preintegration complexes. Integration is more difficult to compare between mouse and human cells. One way to quantify integration of HIV-1 is to separate genomic from small molecular weight DNA and quantify the integrated provirus. This experiment needs to be carefully controlled since a small amount of contamination with un-integrated HIV DNA can influence the results. To measure integration in human cells, many groups have taken advantage of the abundant Alu sequences which are found, on average, every 500bp throughout the genome [44]. SINE B2 sequences in mouse cells are less frequent; however, they can be used to compare the efficiency of HIV-1 integration among mouse cell samples. Because HIV-1 integration cannot be quantitatively compared between species by using these methods, we have chosen to compare integration efficiency between mouse and human by infecting cells with HIV-GFP and using GFP expression as a surrogate marker for integration, an approach used previously to study blocks in the HIV replication cycle in 3T3 cells [15], [16].

We found that Cyclosporin A can double the infection efficiency of primary mouse T cells at a concentration that inhibits NFAT signaling. CsA probably works on steps after reverse transcription, since it had similar effect on cells treated between 12 h and 24 h post-infection (data not shown). It was reported that interruption of the interaction between HIV-1 CA and Cyclophilin A has opposite effects in human cells and non-permissive cells from other species [26], [27], [28]. Thus, CsA accentuated the restriction of Ref1 (Trim5α or other host factors [45]) in human cells, while greatly relieving the restriction of LV-1 in other species. For example, treatment of owl monkey cells with CsA increased the proportion of HIV-1 DNA in the nucleus and led to a 100–1000-fold increase in virus titer [26], [27], [28]. It was reported that Cyclosporin A enhanced the infection efficiency in primary mouse bone marrow cells but had no effect on primary mouse splenic T cells [46]. The failure to detect the enhancement in mouse T cells was likely due to the absence of TCR signals, which obscured the effect of Cyclosporin A (Figure 6B). We speculate that there is a mechanism similar to that in non-human primates, involving Cyclophilin A blockade of mouse T cell infection with HIV-1. It will be interesting to determine whether the G89V HIV mutant, whose CA cannot interact with Cyclophilin A, has a higher infection efficiency in mouse T cells. Mouse cells do not have a Trim5α ortholog, but other related proteins may be involved in this host restriction.

### Activation signals for HIV-1 infection

In the process of optimizing conditions for HIV-1 infection of mouse T cells, we found that additional TCR signals were essential, in a limited time window, for infection of pre-activated T cells. In the absence of a secondary stimulus late reverse transcription products were decreased 2–3 fold. Although this suggests that TCR signals can enhance the reverse transcription of HIV-1, this is not likely to be the critical step affected, since providing TCR signals at the peak of reverse transcription did not result in a rebound of reverse transcription (data not shown), but rescued infection to an average of 70% of the maximum efficiency (Figure 4F). The TCR signals increased the efficiency of HIV-1 integration in mouse T cells about 5–8 fold (Figure 4H). It remains possible that TCR signals also affect steps subsequent to HIV integration. However, similar results were obtained using reporter HIV viruses whose GFP expression was driven by either CMV or LTR. Moreover, there was no change in GFP expression in infected primary mouse T cells when the secondary TCR signal was provided at 5 days instead of 3 hours post infection (data not shown). An accurate assessment will require a reliable technique that can quantify and compare levels of the integrated provirus between primary mouse and human T cells.

In a recent paper describing HIV-1 infection of primary mouse T cells from hCD4/CXCR4 transgenic mice, a significant pre-integration block was reported [43]. The block was ascribed to mis-localization of HIV-1 integrase protein from nucleus to the cytoplasm, but this was assessed in mouse 3T3 cells. We and others [15], [16]] have not observed a pre-integration block in 3T3 cells, suggesting that the localization of integrase protein is unrelated to successful nuclear import or integration. The major difference between our results and those in the recent paper most likely lies in the continued TCR stimulation that we employ, although it remains possible that there is an effect on integrase localization in the primary T cells.

Identification of the TCR signaling pathways involved in relieving the pre-integration blocks may provide further insight towards developing a mouse model for HIV-1 replication. It remains to be determined if antigen-presenting cell-T cell interaction is sufficient to provide the secondary TCR signals. Further experiments must be performed to establish which molecules downstream of the TCR can function to increase HIV infection. PKCθ and CARMA1, which are required for NF-κB activation in T cells, as well as WASp are dispensable for efficient HIV infection after TCR stimulation. However, the PI3K-AKT pathway may be involved in promoting proviral integration [47]. PI3K inhibitor, added 1 h post-infection of primary mouse T cells, completely blocked HIV-1 infection in the presence of anti-CD3/28 and IL-2 (Figure S3). It is known that at different concentrations PI3K inhibitor can block both PI3K and its downstream kinase [48]. Additionally, PI3K is involved in DNA damage repair. Molecules involved in the DNA damage repair pathway have been implicated in HIV-1 integration, although these results are controversial [49], [50], [51]. Our preliminary results thus suggest that the PI3K/Akt, but not the NF-κB, pathway is important in facilitating post-entry steps of HIV replication in murine T cells.

The effect of secondary TCR signaling is not necessary for mouse T cell lines and primary human T cells. This implies that there is a species-specific factor(s) responsible for the pre-integration block in primary mouse T cells. In mouse cell lines, the TCR signals may have been bypassed upon transformation, and hence such stimulation has little effect. We note a much higher infection efficiency found in the mouse T cell lines used in this study, compared to another report indicating significant pre-integration blocks [25]. This may be due to the different genetic backgrounds between cell lines, similar to the observation shown in Figure 4C that primary cells from B6 and Balb/c are less infectable than cells from transgenic or knockout mice with mixed background. Further work needs to be done to identify the specific protein(s) involved in the TCR-dependent increase in HIV-1 infection. This may allow the generation of genetically manipulated mice that are permissive for single round HIV infection and may provide a better understanding of the pre-integration steps in HIV-1 infection.

### Virus release

The last step of the HIV-1 life cycle is virus budding and release. HIV-1 transgenic mice can potentially be used to study this and other post-gene expression steps. However, HIV has been shown to be toxic for CD4^+^ T cells [12], [52], [53], so mature CD4^+^ T cells in the HIV transgenic mice have potentially undergone changes to allow them to survive. For this reason, we assessed particle maturation and release in freshly-infected T cells from hCyclin T1 transgenic mice. Because primary mouse T cells are very sensitive to apoptosis and activated primary CD4^+^ T cells are prone to activation-induced cell death, we used low viral MOI and optimized conditions to avoid cell death. We used VSVG-pseudotyped virus for these assays as the Env protein of HIV-1 was found to be more toxic to primary T cells than VSVG, as indicated by apoptosis analysis based on AnnexinV staining (data not shown). Additionally, removal of CD25^+^CD4^+^ regulatory T cells resulted in substantial increase in survival of CD4^+^ T cells. Under these conditions, we observed a block in release of HIV-1 from mouse cells.

Compared to virus released from human T cells, virus released from primary mouse T cells was 6–10 fold less infectious. Compounding this reduced infectivity of released particles was the dramatic decrease in intracellular Gag. It is possible that viral restriction by host factors is more significant when virus production is low. We will have a better understanding of infectivity of particles released from mouse cells once we have improved the transcription and processing of HIV-1 Gag protein.

Electron microscopy further confirmed the release block in mouse primary T cells. In these experiments, virus budding appeared to be abnormal in mouse T cells. We observed virus particle budding into the nuclear membrane and inducing swelling between the inner and outer nuclear membrane. We failed to observe virus budding out of the plasma membrane. This is in contrast to human cells where we easily observed viral release from the plasma membrane.

In conclusion, we have demonstrated that primary T cells from hCD4/CCR5/Cyclin T1 mice support efficient HIV-1 virus entry, reverse transcription and nuclear import. TCR signals were found to be essential to relieve a pre-integration block in mouse T cells, and this block was further relieved by CsA. The effect on proviral integration was confined to primary T cells, highlighting the importance of studying such cells in addition to immortalized cell lines in our efforts to acquire a better understanding of HIV replication. Our results also indicate that additional host factors wait to be identified to solve inefficient transcription, Gag translation, Gag processing and virus release.

## Materials and Methods

### Mice and Plasmids

Human CD4/CCR5/Cyclin T1 transgenic mice were generated individually and bred into double or triple transgenic lines. Constructs included a human genomic CD4 minigene [54] and human CCR5 and Cyclin T1 cDNAs under the control of murine *Cd4* enhancer and human *CD4* promoter, silencer, and monocyte-lineage enhancer [5], [12]. Phenotype of hCD4 and hCCR5 transgenic mice was assessed by flow cytometry analysis of mouse blood stained with Pacific Blue-mCD4, APC-hCD4 and APC-Cy7-hCCR5 antibodies. hCyclin T1 transgenic mice were identified by PCR analysis of DNA extracted from mouse tails. Primers used were GAATACCGC GCGAAGCAT (forward) and CCTTTTACGAG TAGAACTGGAAGAG (reverse). Wild type Balb/c and C57BL6 mice were purchased from Taconic. All animal experiments were performed in accordance with approved protocols from the NYU Institutional Animal Care and Usage Committee. HIV-CMV-GFP plasmid was made by replacing the *nef* gene in the HIV-HSA vector [55] with *CMV-GFP* utilizing NotI and XhoI sites. CMV-GFP was amplified from pEGFP-C1 vector by PCR using primers as follows: forward ATAAGAATGCGGCCGCTTAATAGTAATCAATTACGG, reverse: CCAGCTCGAGATTACTTGTACAGCTCGTCCATGCCG. pM310 plasmid encoding Vpr-β-lactamase was obtained from Michael D. Miller (Merck). R7/3-GFP and its D116A integrase mutant plasmids were obtained from Michael Topper and Mark Muesing (Aaron Diamond AIDS Research Center) [56]. Plasmid for 2-LTR circles was obtained from Mark Sharley (UMass).

### T cells

Human CD4^+^ T cells were purified from peripheral blood and cord blood obtained from the New York Blood Center. The blood procurement from healthy donors for this study has been reviewed and approved by the New York University School of Medicine Institutional Review Board. Mononuclear cells were isolated by Ficoll-Paque (GE Healthcare) gradient centrifugation. CD4^+^ T cells were further purified by positive selection using anti-hCD4 magnetic microbeads (1∶20 dilution) and MACS column (Miltenyi Biotech), following the manufacturer's protocols. For mouse primary CD4^+^ T cells, single-cell suspensions from spleen and mesenteric lymph nodes were first enriched by staining with PE-conjugated anti-mCD8, mB220, mCD11b, mCD11c, mCD25 (all 1∶100 dilution) and mTer119 (1∶66 dilution), followed by incubation with anti-PE magnetic microbeads and depletion through MACS column. The depleted fraction was positively-selected by anti-mCD4 magnetic microbeads to get >99% pure resting primary mouse CD4^+^ T cells. T cells were cultured in RPMI 1640 (Invitrogen) supplemented with 10% heat-inactivated Fetal Calf Serum (Hyclone), 2 mM L-glutamine, 100 u/ml penicillin, 100 µg/ml streptomycin, and 50 µM β-mercaptoethanol. Cells were plated at a density of 1–1.2×10^6^ per well of a 24-well culture plate.

### Virus stocks

HIV-1 stocks were generated by calcium phosphate transfection of 2.5×10^6^ 293T cells in 10 cm plate with 15 µg virus vector (JRCSF, HIV-GFP, HIV-CMV-GFP, R7/3-GFP or its D116A mutant) and 5 µg Env vector (JRCSF or VSVG) as indicated by envelope pseudotyping. Virus supernatants were collected 48 h post transfection and treated with Benzonase (15U/ml, EMD Bioscience) in 1x buffer (50 mM Tris-HCl pH 8.0, 1 mM MgCl_2_, and 0.1 mg/ml BSA) for 15 min at 37°C. Aliquots of virus were frozen at −80°C until use. Virus titer was determined by serial dilutions on GHOST/CCR5 or GHOST/parental cell lines in the presence of polybrene (8 µg/ml). R7/3 GFP and its D116A mutant viruses were also titrated with reverse transcriptase assay (RT assay kit, Roche) to ensure that similar amounts of virus were used for infection in Figure 3A for detecting 2LTR signals. Flow cytometry analysis was done 36 h after infection of GHOST cells to assess percentage of GFP positive cells. MLV was produced by transfection of Phoenix E cells with MSCV retrovirus vector expressing Cre-GFP. Titer was titrated on 3T3 cells.

### T cell activation and infections

For human T cell analysis, CD4^+^ T cells were cultured with PHA (5 µg/ml, Sigma) and hIL-2 (30 Unit/ml, Roche) for two days. Cells were washed twice with PBS and cultured in hIL-2 alone for 1 day before infection. For human cord blood T cells, CD4^+^ T cells were activated by cross-linking with plate-bound anti-CD3/CD28 antibody (5 µg/ml each, eBioscience). The plates were first coated with goat anti-mouse IgG (10 µg/ml, Caltag). For mouse T cell analysis, CD4^+^ T cells were cultured on anti-CD3/28 (clone 145-2C11 for anti-CD3, clone 37N for anti-CD28, Memorial Sloan-Kettering Cancer Institute, 5 µg/ml each, 500 µl per well in 24-well-plates coated at 37°C for 2–3 h) for 36 h before infection. Virus was added to cells together with polybrene (8 µg/ml). Spin-infection was done by centrifugation at 2,000 rpm for 1 h at R.T. Cells with virus were incubated at 37°C for 2 additional hours and then washed twice to remove virus. Human or mouse IL-2 (30 Unit/ml, eBioscience) was added and maintained all the time after infection. 100 unit/ml of mIL-2 was used during the activation and culture of CD4^+^ T cells purified from *Pkcθ* ^−/−^ and *Carma1* ^−/−^ mice.

### Flow Cytometry

Flow cytometric analysis was performed on an LSR II cytometer (BD Biosciences) and analyzed using FlowJo software (Tree Star Inc.). Antibodies used in this study were obtained through eBioscience or BD Biosciences.

### Entry assay

Viruses containing Vpr-β-lactamase were generated by co-transfection of 10 µg pM310 with 15 µg JRCSF or 15 µg HIV-GFP (Env^−^) plus 3 µg VSVG into 293T cells in 10 cm plates using the calcium phosphate method. Virus containing supernatants were concentrated by ultracentrifugation at 25,000 rpm for 1.5 h at 4°C and resuspended in 200 µl of RPMI medium. Virus titer was determined on GHOST cell lines [57]. For entry assay, 0.5–1×10^5^ cells per well in 96-well-plate were spin-infected with different MOIs of virus for 1 h and incubated for another 2 h at 37°C to ensure complete virus entry. Cells were washed once with CO_2_-independent medium. Cells in each well were provided with 30 µl loading reagent (6x, Protocol for Beta-lactamase loading solutions, Invitrogen) and 150 µl CO_2_-independent medium supplemented with 10% serum and IL-2. Cells were incubated at 18–20°C for 2 h for mouse T cells and overnight for human T cells. After incubation, cells were washed with CO_2_-independent medium and fixed with PFA. Entry efficiency was analyzed by flow cytometry using FITC and DAPI channels, for un-cleaved substrate and cleaved product respectively. Virus without Vpr-β-lactamase was used for gating control. R5 inhibitor TAK-779 (1 µM) [58] was used to confirm R5-dependent entry.

### RT assay

5×10^5^ mouse CD4^+^ T cells were activated with anti-CD3/28 in 48-well-plates for 36 h. HIV-GFP (VSVG pseudotyped, MOI = 2, benzonase treated) was used for spin-infection. Cells were collected at 0, 1, 2, 3, 6, 9, 12, 15 and 24 h post-infection, washed with PBS and frozen at –80°C. As a control, AZT (15 µM) was added at different time points and samples were collected starting from the next time-point. Total DNA was extracted by DNeasy kit (Qiagen) or phenol and dissolved in 50 µl AE buffer (Qiagen). Real-time PCRs were done using primers and probes specific for U5, 2^nd^ transfer and 2-LTR circles [59], [60], Serial dilutions of HIV-GFP or 2-LTR plasmid in genomic DNA were used to generate a standard curve in each plate. Calculation of cell number was determined based on real-time PCR for gene encoding eukaryotic18S rRNA (Hs99999901_s1, Taqman, Applied Biosystem) with common sequence for human and mouse. Real-time PCRs were performed using QuantiTect Multiplex PCR mix (Qiagen) in the i-Cycler Sequence Detection System (Bio-Rad). The running conditions and primer sequences were as the follows. For 2-LTR circles, 95°C 5 min, then 95°C 45 s, 55°C 1 min, 72°C 1 min for 40 cycles. All others, 95°C 5 min, then 95°C 45 s, 60°C 1 min for 40 cycles. Primer sequences for U5: Forward TCTCTGGTTAGACCAGATCTG, Reverse GTCTGAGGGATCTCTAGTTAC, probe FAM-AGCCTCAATAAAGCT TGCCTTGAGTGC-TAMRA. Primers for 2^nd^ strand transfer: Forward TTTTAGTCAGTGTGGAAAATCTGTAGC, reverse TACTCACCAGTCGCCGCC probe: FAM-TCGACGCAGGACTCGGCTTGCT-TAMRA. Primers for 2-LTR: Forward, TAGACCAGATCTGAGCCTGGGA, reverse, GTAGTTCTGC CAATCAGGGAAG, probe FAM-AGCCTCAATAAAGCTTGCCTTGAGTGC-TAMRA. To quantify copy numbers of integrated provirus, extracted DNAs were subjected to a first-round PCR to amplify integrated HIV signals using primers specific for HIV-1 and retrotransposon B2. Primer sequences were: B2-1: TTCACAACTCTCGGT GGATGGTGG; B2-2: GACCTTTGGAAGAGCAGTCG and HIV-1 primers: NL750RC: TACTCACCAGTCGCCGCC; NL800R: GAGCGTGGGTAGAGAGAGGAAG. Samples were denatured at 95°C for 3 min, followed by 30 cycles at 95°C for 45 s, 55°C for 1 min, and 72°C for 3 min. Products generated from this first round of amplification were diluted in water (1:1,200) and subjected to real-time PCR using primers and probe specific for HIV-1 U5.

### Viral RNA analysis

Total RNA was purified using Qiagen RNeasy kit from cells infected with HIV-GFP (VSVG) for 2 d. cDNAs were generated from RNA by RNase H^−^reverse transcriptase (Invitrogen) using random primers. Real-time PCR was done using primers specific for U5 and 18S rRNA. As control, no reverse transcriptase was added at the time of cDNA preparation.

### Gag protein analysis

Cells were infected with HIV-GFP (VSVG) in the presence or absence of AZT (10 µM). One day post infection, cells were treated with 0.01–0.04% pronase to remove the virus stuck to the plasma membrane. Two days following infection, cell pellets and supernatants were separated after centrifugation (2,000x g, 5 min) and frozen at −80°C. Cells were lysed in RIPA buffer (10 mM Tris, 150 mM NaCl, 0.1% SDS, 1% Triton, 1% Deoxycholate, 5 mM EDTA). p24 in cell lysates and supernatants was measured by ELISA. ELISA was set up using primary and secondary p24 antibody purchased from CLINIQA. Recombinant HIV-1 p24 was included for standard curves. For western blots, aliquots of cell lysates were separated on 12% acrylamide gradient gels and transferred to PVDF membrane. Western blots were probed with a monoclonal antibody to HIV-1 p24 (NIH AIDs Research and Reference Reagent Program). Membranes were stripped and re-probed with anti-tubulin (Sigma, antibody recognizes both human and mouse tubulin) as loading control.

### Infectivity assays

Infectious HIV-1 in supernatants was quantified by using the Tzmbl cell line [38]. Briefly, Tzmbl cells (HeLa cells expressing hCD4, CXCR4 and CCR5, NIH AIDS reagents) were seeded in 48-well plates and inoculated with HIV-1-containing supernatant, which was serially diluted to make sure the infection was not saturated. AZT and TAK-779 were added at 16 h post-infection to prevent secondary infection. Thirty-six hours later, the cells were lysed and firefly luciferase activity was determined (Promega).

### Electron Microscopy

Human or mouse T cells were infected with HIV-GFP (VSVG) at MOI of 2. For human cells, 3 d after infection, cells were pelleted and fixed. For mouse T cells, cells were FACS sorted for GFP*^+^* cells and allowed to recover overnight at 37°C. Cells were resuspended in 1 ml of PBS, transferred to 1.5 ml Eppendorf tubes and centrifuged at 800×g for 1 minute. Pellets were fixed in 2.5% glutaraldehyde for 2 h at 4°C and post-fixed with 1% osmium tetroxide for 1.5 h at room temperature, then processed in a standard manner and embedded in Embed 812 (Electron Microscopy Sciences, Hatfield, PA). Semi-thin sections were cut at 1 µm and stained with 1% Toluidine Blue to evaluate the quality of preservation. Ultrathin sections (60 nm) were cut, mounted on formover coated copper grids and stained with uranyl acetate and lead citrate by standard methods. Stained grids were examined using a Philips CM-12 election microscope and photographed with a Gatan (1k×1k) digital camera.

## Supporting Information

Figure S1 VSVG mediates efficient entry into mouse and human T cells. Entry assay for VSVG-pseudotyped HIV-1 containing Vpr-β-lactamase at MOI of 0.04 was performed as described in the legend to Figure 1.
(0.90 MB EPS)

Figure S2 Benzonase treatment did not affect the infectivity of HIV-1. Dilutions of the viruses before and after benzonase treatment were added to Tzmbl reporter cells. Firefly luciferase assays were done at 48 h post-infection.
(0.64 MB EPS)

Figure S3 PI3K mediated pathways are important for HIV-1 infection in primary mouse T cells. CD4+ T cells from hCyclin T1 transgenic mice were activated with anti-CD3/28 and infected with HIV-GFP (VSVG). PI3K inhibitors wortmannin and Ly294002 were added at the time of infection at concentrations indicated and removed at 24 h post infection. DMSO was used as the control. Infection efficiency was assayed by FACS for GFP expression at 48 h post infection.
(0.66 MB EPS)
